# Hemiurid and lecithasterid digenean trematodes and camallanid and cucullanid nematodes parasitizing flounders collected off the coast of Rio de Janeiro State, Brazil

**DOI:** 10.1590/S1984-29612022011

**Published:** 2022-03-11

**Authors:** Michelle Cristie Gonçalves da Fonseca, Nilza Nunes Felizardo, Eduardo José Lopes Torres, Delir Corrêa Gomes, Marcelo Knoff

**Affiliations:** 1 Laboratório de Helmintos Parasitos de Vertebrados, Instituto Oswaldo Cruz, Fundação Oswaldo Cruz – FIOCRUZ, Rio de Janeiro, RJ, Brasil; 2 Laboratório de Inspeção e Tecnologia de Pescado, Universidade Federal Fluminense – UFF, Niterói, RJ, Brasil; 3 Laboratório de Helmintologia Romero Lascasas Porto, Faculdade de Ciências Médicas, Universidade do Estado do Rio de Janeiro – UERJ, Rio de Janeiro, RJ, Brasil

**Keywords:** Hemiuridae, Lecithasteridae, Camallanidae, Cucullanidae, flounders, Brazil, Hemiuridae, Lecithasteridae, Camallanidae, Cucullanidae, linguados, Brasil

## Abstract

A total of 132 flounder specimens (60 *Paralichthys isosceles*, 36 *Paralichthys patagonicus* and 36 *Xystreurys rasile*) were collected off the coast of the state of Rio de Janeiro, Brazil. The fish were measured, necropsied, and had their organs investigated for hemiurid and lecithasterid digenean trematodes and camallanid and cucullanid nematodes. Taxonomic identification of the parasites was based on morphological and morphometric characters and was conducted using bright-field and scanning electron microscopies. The trematodes *Lecithochirium monticellii* and *Aponurus laguncula* were found parasitizing *P*. *isosceles*, *P. patagonicus* and *X*. *rasile* while the nematodes *Procamallanus* (*Spirocamallanus*) *halitrophus* and *Cucullanus bonaerensis* were found parasitizing *P*. *isosceles* and *X*. *rasile* and *P*. *isosceles*, *P. patagonicus* and *X*. *rasile*, respectively. Parasite indices of prevalence, mean intensity, mean abundance, and range of infection, as well as infection site, were evaluated for each parasite species. This study allowed to evidence the first occurrence of *P. patagonicus* by *L. monticellii*; *X. rasile* by *A. laguncula* and *P*. (*S*.) *halitrophus*; and *P. isosceles* and *P. patagonicus* by *C. bonaerensis* in the Western South Atlantic Ocean.

## Resumo

Um total de 132 espécimes de linguados (60 *Paralichthys isosceles*, 36 *Paralichthys patagonicus* e 36 *Xystreurys rasile*) foram coletados na costa do Estado do Rio de Janeiro, Brasil. Os peixes foram medidos, necropsiados e tiveram seus órgãos investigados para o encontro de trematodeos digenéticos, das famílias Hemiuridae e Lecithasteridae, e nematoides das famílias Camallanidae e Cucullanidae. A identificação taxonômica dos parasitos foi baseada nos caracteres morfológicos e morfométricos, usando-se as microscopias de campo claro e eletrônica de varredura. Os trematódeos, *Lecithochirium monticellii* e *Aponurus laguncula*, foram encontrados parasitando *P*. *isosceles*, *P. patagonicus* e *X*. *rasile*, enquanto os nematoides, *Procamallanus* (*Spirocamallanus*) *halitrophus* e *Cucullanus bonaerensis*, foram encontrados parasitando *P*. *isosceles* e *X*. *rasile* e *P*. *isosceles*, *P. patagonicus* e *X*. *rasile*, respectivamente. Os índices parasitários de prevalência, intensidade média, abundância média e amplitude de variação da intensidade de infeção, bem como os sítios de infecção foram avaliados para cada espécie de parasito. Este é o primeiro registro de *P. patagonicus* por *L. monticellii*; *X. rasile* por *A. laguncula* e *P*. (*S*.) *halitrophus*; e de *P. isosceles* e *P. patagonicus* por *C. bonaerensis* no Oceano Atlântico Sul Ocidental.

## Introduction

The flounder species *Paralichthys isosceles* Jordan, 1890, *P. patagonicus* Jordan, 1889 and *Xystreurys rasile* (Jordan, 1891) (Paralichthyidae) represent important primary fishery resources in the coastal waters of Brazil (Figueiredo & Menezes, 2000). According to Cerqueira et al. (1997), Massa et al. (2005) and Bernardes et al. (2005), the flounder fishery is referred to as “fine fishing” due to high commercial interest, meat quality and market price and because of broad sale in domestic and foreign markets.

Digenean trematodes present a certain degree of specificity in relation to their site of infection and their definitive host, although some species may infect different sites, such as adult individuals of species of the families Hemiuridae and Lecithasteridae when parasitizing the digestive system of fish (Eiras et al., 2006).

Parasitic nematodes are important pathogens associated with human and non-human animal health (Acha & Szyfres, 2003). Nematodes inhabit hosts of fresh, brackish and marine waters around the world, and some are known to be agents of serious fish diseases that cause considerable losses to the fishing industry (Klimpel & Palm, 2011). Adult nematodes of the families Camallanidae and Cucullanidae are commonly found parasitizing the intestine of various fish, their definitive hosts (Williams & Jones, 1994; Lanfranchi et al., 2004; Cárdenas & Lanfredi, 2005).

Studies, including morphological and ecological surveys, have reported some groups of helminths parasitizing the flounder species *P*. *isosceles*, *P*. *patagonicus* and *X*. *rasile* (Paralichthyidae) in Brazil (Felizardo et al., 2009a, b, 2010, 2011, 2018; Fonseca et al., 2012, 2016, 2019; Knoff et al., 2012; Alarcos et al., 2016; Eiras et al., 2016; Justo et al., 2017) including species of trematodes and nematodes. The present paper continues these studies on flounders occurring off of the Brazilian coast. Thus, aiming to continue the study of these helminths was investigated the presence of hemiurid and lecithastherid digenean trematodes and camallanid and cucullanid nematodes parasitizing paralichthyid flounders from off the coast of the state of Rio de Janeiro, Brazil. These helminths were identified morphologically and morphometrically by bright-field and scanning electron microscopies, calculated their parasitic indices and determined their sites and ranges of infection.

## Material and Methods

### Host fish and parasite sampling

One hundred and thirty-two flounders were acquired: 60 *P*. *isosceles,* mean length 37.5 cm (16.4-68.9 cm), mean weight 39.1 g (25.0-42.0 g); 36 *P. patagonicus,* mean length 39.2 cm (28.5-59.0 cm), mean weight 747.3 g (280.0-2530.0 g); and 36 *X. rasile,* mean length 29.7 cm (14.0-51.0 cm) and mean weight 371.3 g (25.0-1440.0 g). The fish were obtained in small markets selling only fish caught offshore of the municipalities of Cabo Frio (22º52'46” S, 42º01'07” W), Niterói (22º53'00” S, 43º06'13” W), Rio de Janeiro (22°54'13” S, 43°12'35” W), and Angra dos Reis (23º00'24” S, 44º19'05” W), of the state of Rio de Janeiro (RJ), Brazil. Fish were transported on ice to the laboratory to investigate the presence of helminths. The fish were identified according to Nakamura et al. (1986) and Figueiredo & Menezes (2000). After necropsy, internal organs were placed in Petri dishes with 0.65% NaCl solution to evaluate the presence of hemiurid and lecisthasterid trematodes and camallanid and cucullanid nematodes. Trematodes were fixed in AFA (alcohol, formalin, and acetic acid), preserved in 70% ethanol, stained in Langeron´s carmine or in Harris’ hematoxylin, clarified in beechwood creosote, and preserved as whole mounts on Canada balsam. Nematodes were fixed in AFA, preserved in 70% ethanol and later clarified with Amman's lactophenol (Knoff & Gomes, 2012).

### Morphological identification and parasitic indices

Hemiurid species were identified using Looss (1908), lecithasterid species using Skrjabin & Guschanskaja (1955), camallanid species using Fusco & Overstreet (1978) and Cárdenas & Lanfredi (2005) and cucullanid species using Lanfranchi et al. (2004). The taxonomic classification of digenean trematodes was according to Gibson et al. (2002) while that of nematodes followed De Ley & Blaxter (2004). Samples were analyzed using an Olympus BX-41 bright-field microscope and images captured using a Canon Power Shot A640 digital camera coupled to a Zeiss Axiophot microscope using a Nomarski’s differential interference contrast (DIC) apparatus. Illustrations were made using a drawing tube connected to the microscope. Measurements are provided in millimeters (mm) unless otherwise indicated, with averages in parentheses. Scanning electron microscopy (SEM) was used to elucidate topographic details of specimens of *Cucullanus*, with processing according to Lopes Torres et al. (2013). Samples for SEM were fixed in Karnovsky solution, dehydrated in an ethanol series (70-100%), CO_2_ critical-point dried, coated in gold and examined and photographed using JEOL SM - 25 SII scanning electron microscope under an acceleration voltage of 15 kV. The parasitic indices of prevalence (P), mean intensity (MI) and mean abundance (MA) were calculated according to Bush et al. (1997). Range of infection (RI) and sites of infection (SI) are also presented. Representative specimens were deposited in the Helminthological Collection of the Oswaldo Cruz Institute (CHIOC), FIOCRUZ, Rio de Janeiro, RJ, Brazil.

## Results

A total of 1,127 parasite specimens were collected from the sampled flounders, with 783 digenean trematodes of two species of two families (398 Hemiuridae, 385 Lecithasteridae) and 344 nematodes of two species of two families (46 Camallanidae, 298 Cucullanidae). The total number of helminths per flounder species were: 401 from *P*. *isosceles*, 584 from *P*. *patagonicus* and 142 from *X*. *rasile*. The species taxonomic identifications follow.

Platyhelminthes Minot, 1876, Rabditophora Ehlers, 1985, Neodermata Ehlers, 1985, Trematoda Rudolphi, 1808, Digenea Carus, 1863, Hemiuroidea Looss, 1899, Plagiorchiida La Rue, 1957, Hemiurata, Skrjabin & Guschanskaja, 1954, Hemiuroidea, 1899

Hemiuridae Looss, 1899

Lecithochiriinae Lühe, 1901

*Lecithochirium* Lühe, 1901

*Lecithochirium monticellii* (Linton, 1898) Skrjabin & Guschankaja, 1955

(Figures 1 and 2)

**Figure 1:**
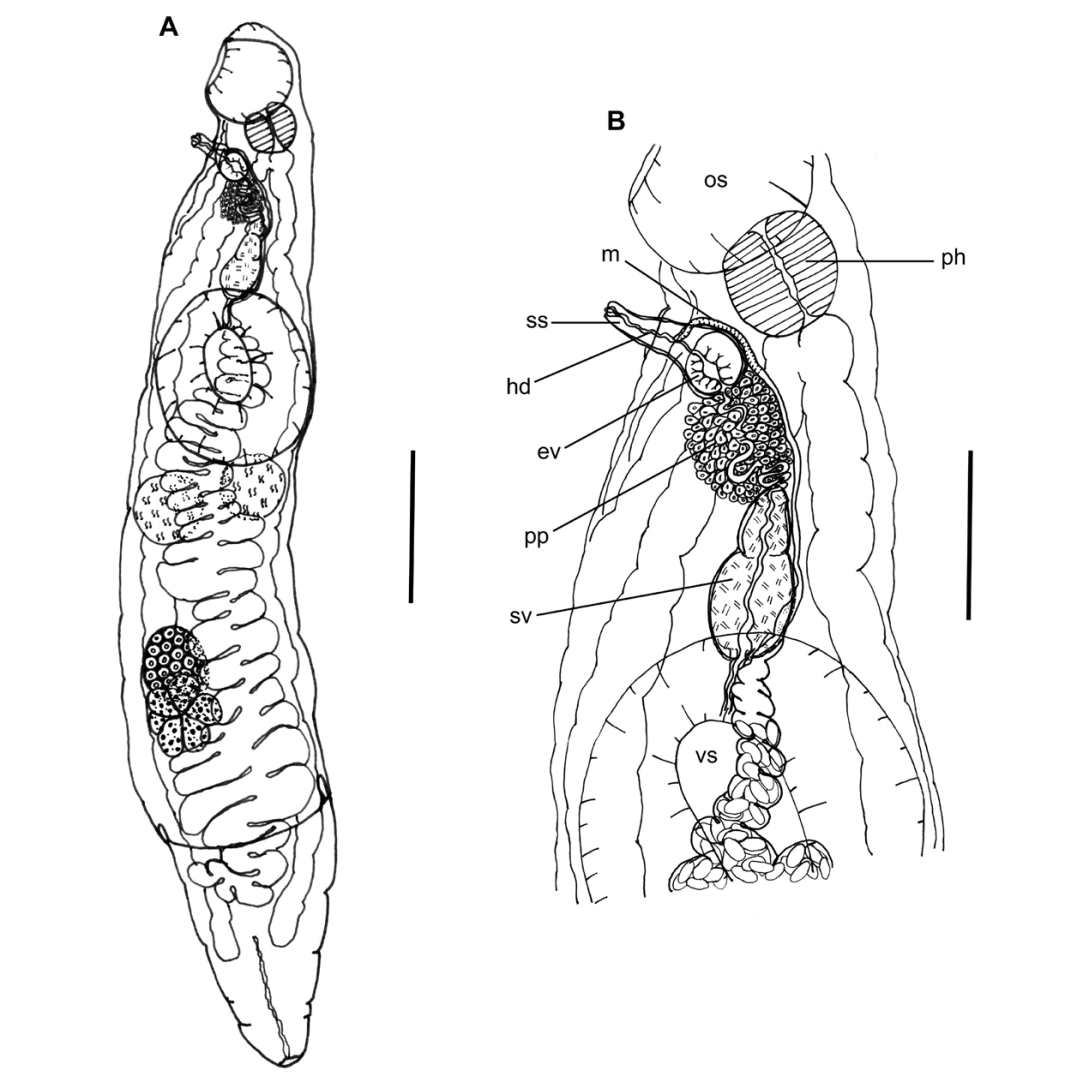
*Lecitochirium monticellii* collected from *Paralichthys patagonicus*. (A) Entire worm, ventrolateral view; (B) Detail of terminal genitalia. Abbreviations: ev = ejaculatory vesicle, hd = hermaphroditic duct, m = metraterm, os = oral sucker, pp = pars prostatica, ph = pharynx, sv = seminal vesicle, ss = sinus sac, and vs = ventral sucker. Bars A = 0.2 mm, B = 0.1 mm.

**Figure 2:**
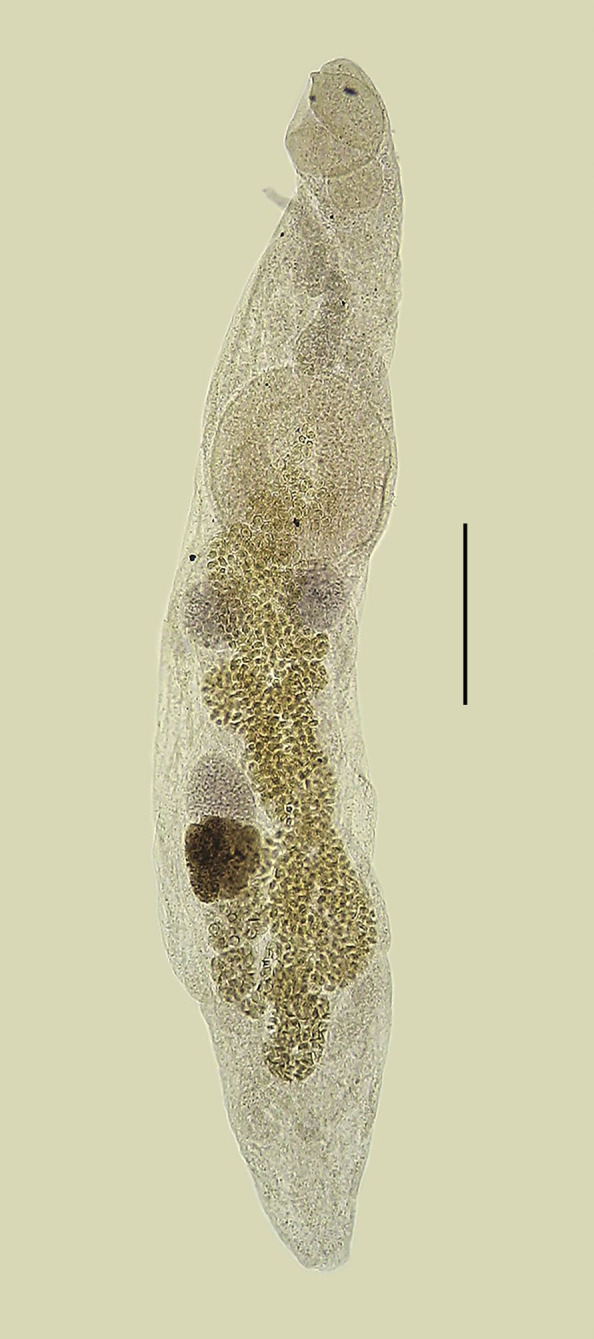
*Lecitochirium monticellii* collected from *Paralichthys patagonicus*. Entire worm, ventrolateral view. Bar = 0.2 mm.

Features observed in 35 specimens, 11 from *P*. *isosceles*, 13 from *P*. *patagonicus* and 11 from *X*. *rasile*. Body elongate, subcylindrical, with smooth tegument. Preoral lobe present. Ecsoma well developed, variable in length, retracted in some specimens. Oral sucker subglobular, subterminal. Ventrocervical groove present, with thickened walls. Pharynx subglobular, smaller than oral sucker, partly overlying oral sucker. Ventral sucker subglobular, developed, larger than oral sucker, pre-equatorial. Ceca lined with thick epithelium, entering ecsoma. Testes two, not lobed, rounded, smooth, variable in shape, tandem, lateral or diagonal, in larger specimens in distant zones, in smaller specimens the posterior portion of the anterior testis coincides with the anterior portion of the posterior testis, margins often obscured by uterine loops. Vas efferens uniting to vas deferens close to medial margin of ventral sucker. Vas deferens, small duct, uniting to seminal vesicle. Seminal vesicle tripartite, partially overlapping anterior margin of ventral sucker, with thick wall, posterior portion largest, anterior portion smallest, uniting to ejaculatory duct. Ejaculatory duct, small, slightly sinuous, sometimes swollen in its proximal, median or even distal portion, appearing as an external ejaculatory vesicle, surrounded by numerous prostatic cells, forming a well-developed pars prostatica. Ejaculatory duct uniting to sinus sac meeting the ejaculatory vesicle. Sinus sac ‘separogermiductus-type’, oval, well-developed, thick walled, containing well-developed ejacutatory vesicle (sometimes described as prostatic vesicle), and long, muscular, protrusible hermaphrodict duct opening in a genital pore. Genital pore medial, at or slightly posterior to pharyngeal level. Ovary rounded, smooth, post-testicular, intercecal, in mid-hindbody or more posterior, at some distance from posterior testis, in same field as testes but in separate zones, margins often obscured by uterine loops. Uterus descending into postvitelline space, reaching ecsoma and filling most of hindbody, metraterm entering sinus sac ventrally, joining male duct slightly anterior to ejaculatory vesicle. Vitellaria paired, contiguous, overlapping posterior portion of ovary, one trilobed and one quadrilobed with lobes somewhat longer than wide. Eggs small, operculate, numerous. Excretory pore terminal, excretory duct bifurcating posterior to ventral sucker, arms unite dorsal to oral sucker.

Morphometrics shown in Table 1.

**Table 1:** Morphometric data of *Lecithochirium monticellii* collected from *Paralichthys isosceles*, *P. patagonicus* and *Xystreurys rasile* off the coast of the state of Rio de Janeiro, Brazil.

	Paralichthys isosceles	Paralichthys patagonicus	Xystreurys rasile
	(n = 11)	(n = 13)	(n = 11)
Body (L)	0.52-2.83 (1.19)	0.72-1.55 (1.16)	0.80-1.32 (1.00)
Body (W)	0.06-0.96 (0.28)	0.12-0.27 (0.21)	0.12-0.27 (0.17)
Pharynx (L)	0.03-0.07 (0.05)	0.03-0.07 (0.04)	0.03-0.06 (0.04)
Pharynx (W)	0.03-0.07 (0.05)	0.03-0.07 (0.04)	0.03-0.04 (0.03)
Oral sucker (L)	0.06-0.12 (0.10)	0.06-0.12 (0.07)	0.04-0.08 (0.05)
Oral sucker (W)	0.06-0.13 (0.09)	0.06-0.14 (0.08)	0.05-0.17 (0.68)
Ventral sucker (L)	0.11-0.37 (0.26)	0.14-0.27 (0.19)	0.09-0.23 (0.16)
Ventral sucker (W)	0.10-0.37 (0.22)	0.13-0.25 (0.18)	0.10-0.20 (0.14)
Sinus sac (L)	0.04-0.08 (0.05)	0.03-0.05 (0.04)	0.05-0.07 (0.06)
Seminal vesicle (L)	0.10-0.31 (0.16)	0.10-0.13 (0.12)	0.08-0.17 (0.13)
Seminal vesicle (W)	0.03-0.17 (0.07)	0.05-0.06 (0.05)	0.01-0.06 (0.02)
Anterior testicle (L)	0.04-0.31 (0.11)	0.05-0.12 (0.09)	0.06-0.10 (0.08)
Anterior testicle (W)	0.05-0.19 (0.11)	0.07-0.11 (0.08)	0.06-0.11 (0.08)
Posterior testicle (L)	0.05-0.25 (0.12)	0.06-0.13 (0.10)	0.07-0.13 (0.09)
Posterior testicle (W)	0.05-0.19 (0.11)	0.06-0.12 (0.09)	0.06-0.11 (0.08)
Ovary (L)	0.05-0.25 (0.10)	0.06-0.12 (0.08)	0.05-0.11 (0.07)
Ovary (W)	0.04-0.18 (0.11)	0.05-0.14 (0.10)	0.06-0.13 (0.07)
Viteline gland (L)	0.06-0.27 (0.13)	0.08-0.31 (0.18)	0.08-0.25 (0.18)
Viteline gland (W)	0.04-0.20 (0.12)	0.04-0.21 (0.13)	0.07-0.21 (0.13)
Ecsoma (L)	0.10-0.36 (0.23)	0.11-0.35 (0.19)	0.09-0.24 (0.15)
Ecsoma (W)	0.05-0.25 (0.12)	0.07-0.13 (0.12)	0.08-0.12 (0.10)
Eggs (L)*	12.50-27.50 (19.31)	12.50-35.00 (16.60)	12.50-27.50 (19.26)
Eggs (W)*	7.50-17.50 (10.58)	7.50-20.00 (11.75)	10.00-15.00 (11.37)

Measurements are in millimeters, means in parentheses. L = length; W = width; n = number of measured specimens.

* Measurements in micrometers and number of measured eggs = 50.

Hosts: *P*. *isosceles*, *P*. *patagonicus* and *X*. *rasile*

Parasitic indices: P = 33.33%, MI = 4.20 (± 4.93), MA = 1.40 (± 13.43), RI = 1-32 (*P. isosceles*); P = 75.00%, MI = 10.85 (± 15.35), MA = 8.13 (± 18.38), RI = 1-83 (*P. patagonicus*); P = 11.11%, MI = 5.25 (± 1.90), MA = 0.58 (± 2.12), RI = 2-9 (*X. rasile*).

Infection sites: stomach (*P. isosceles, P. patagonicus* and *X. rasile*).

Collected specimens: 84 in *P. isosceles*, 293 in *P. patagonicus* and 21 in *X. rasile*.

Deposited specimens: CHIOC 39206, 39207, 39208, 39209 (*P. isosceles*), CHIOC 39201a-b, 39202, 39203 (*P. patagonicus*) and CHIOC 39204a-b, 39205a-b (*X. rasile*).

Lecithasteridae Odhner, 1905

Lecithasterinae Odhner, 1905

*Aponurus* Looss, 1907

*Aponurus laguncula* Looss, 1907

(Figures 3 and 4)

**Figure 3:**
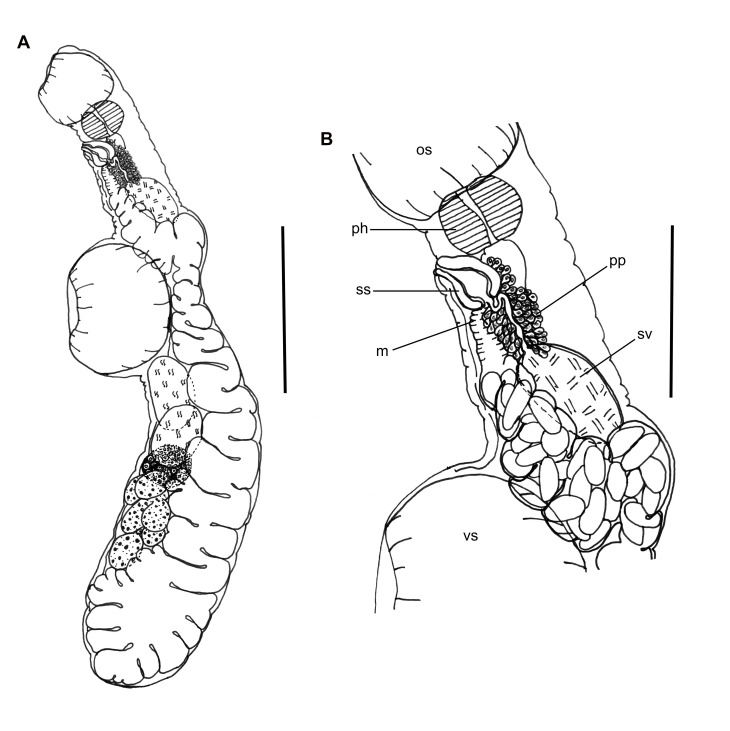
*Aponurus laguncula* collected from *Paralichthys isosceles*. (A) Entire worm, ventrolateral view; (B) Detail of terminal genitalia. Abbreviations: m = metraterm, os = oral sucker, pp = pars prostatica, ph = pharynx, sv = seminal vesicle, ss = sinus sac, and vs = ventral sucker. Bars A = 0.2 mm, B = 0.1 mm.

**Figure 4:**
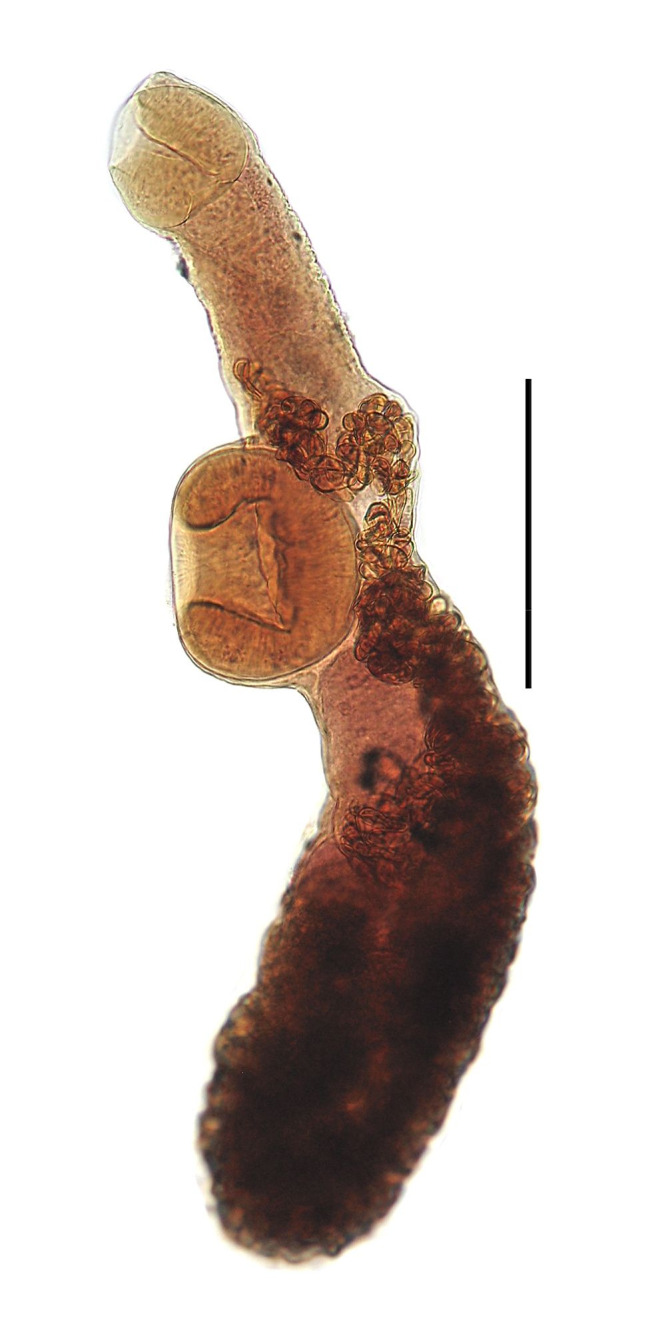
*Aponurus laguncula* collected from *Paralichthys patagonicus*. Entire worm, ventral view. Bar = 0.2 mm.

Features observed in 31 specimens, 10 from *P*. *isosceles*, 13 from *P*. *patagonicus* and 8 from *X*. *rasile*: Body small, narrow, widest near posterior extremity. Tegument smooth. Pre-oral lobe small or not evident. Oral sucker subglobular, subterminal. Ventral sucker subglobular; immediately anterior to mid-body. Pharynx oval. Excretory system generally obscured by eggs, pore terminal, arms unite dorsal to oral sucker. Testes oval, oblique, in anterior hindbody. Seminal vesicle subglobular, in posterior forebody, near anterior margin of ventral sucker. Pars prostatica narrow, curved, with gland cells surrounding external ejaculatory duct. Sinus-sac oval, thin-walled. Genital pore medial. Ovary oval to sub-triangular, posterolateral to posterior testis and posterior to anterior testis. Seminal receptacle inconspicuous. Uterus in mature worms fills most of hindbody, also in median region dorsal to ventral sucker region and up to about middle of forebody, metraterm entering sinus sac ventrally joining male duct immediately at the beginning of sinus sac at the internal ejaculatory duct. Vitellaria paired, seven irregular follicles, one trilobed and one quadrilobed with follicles somewhat longer than wide, overlapping posterior portion of ovary. Eggs numerous, thin-shelled and operculate.

Morphometrics shown in Table 2.

**Table 2:** Morphometric data of *Aponurus laguncula* collected from *Paralichthys isosceles*, *P. patagonicus* and *Xystreurys rasile* off the coast of the state of Rio de Janeiro, Brazil.

	Paralichthys isosceles	Paralichthys patagonicus	Xystreurys rasile
	(n = 10)	(n = 13)	(n = 8)
Body (L)	1.17-2.10 (1.45)	0.67-1.75 (1.10)	0.50-0.87 (0.76)
Body (W)	0.22-0.42 (0.31)	0.10-0.45 (0.30)	0.10-0.20 (0.15)
Pharynx (L)	0.02-0.05 (0.04)	0.03-0.08 (0.04)	0.03-0.05 (0.03)
Pharynx (W)	0.02-0.06 (0.05)	0.03-0.08 (0.05)	0.03-0.05 (0.04)
Oral sucker (L)	0.08-0.17 (0.10)	0.06-0.14 (0.09)	0.06-0.08 (0.07)
Oral sucker (W)	0.07-0.17 (0.09)	0.05-0.25 (0.10)	0.06-0.10 (0.08)
Ventral sucker (L)	0.09-0.19 (0.15)	0.11-0.28 (0.21)	0.12-0.14 (0.13)
Ventral sucker (W)	0.08-0.16 (0.13)	0.09-0.27 (0.20)	0.09-0.15 (0.12)
Sinus sac (L)	0.02-0.04 (0.03)	0.04-0.06 (0.05)	0.02-0.07 (0.04)
Seminal vesicle (L)	0.07-0.12 (0.09)	0.07-0.21 (0.13)	0.06-0.11 (0.09)
Seminal vesicle (W)	0.03-0.09 (0.05)	0.04-0.14 (0.07)	0.04-0.05 (0.04)
Right testicle (L)	0.09-0.16 (0.12)	0.06-0.12 (0.08)	0.05-0.10 (0.08)
Right testicle (W)	0.07-0.15 (0.11)	0.05-0.12 (0.09)	0.05-0.11 (0.07)
Left testicle (L)	0.08-0.14 (0.11)	0.06-0.12 (0.09)	0.05-0.10 (0.07)
Left testicle (W)	0.08-0.12 (0.10)	0.05-0.15 (0.10)	0.05-0.10 (0.08)
Ovary (L)	0.08-0.15 (0.09)	0.06-0.13 (0.08)	0.04-0.08 (0.06)
Ovary (W)	0.08-0.14 (0.09)	0.06-0.12 (0.08)	0.05-0.09 (0.06)
Viteline gland (L)	0.15-0.25 (0.18)	0.07-0.47 (0.23)	0.06-0.15 (0.10)
Viteline gland (W)	0.10-0.27 (0.17)	0.06-0.38 (0.21)	0.07-0.14 (0.10)
Eggs (L)*	20.00-35.00 (28.75)	20.00-47.50 (33.48)	12.50-37.50 (27.56)
Eggs (W)*	12.50-15.00 (13.90)	12.50-22.50 (17.26)	7.50-30.00 (14.60)

Measurements are in millimeters, means in parentheses. L = length; W = width; n = number of measured specimens.

* Measurements in micrometers and number of measured eggs = 50.

Hosts: *P*. *isosceles*, *P*. *patagonicus* and *X*. *rasile*

Parasitic indices: P = 36.66%, MI = 6.22 (± 5.11), MA = 2.28 (± 14.84), RI = 2-31 (*P*. *isosceles*); P = 52.77%, MI = 9.78 (± 7.94), MA = 5.16 (± 5.65), RI = 1-25 (*P*. *patagonicus*); P = 11.11%, MI = 15.50 (± 7.18), MA = 1.72 (± 2.12), RI = 1-39 (*X*. *rasile*).

Infection sites: stomach (*P. isosceles, P. patagonicus* and *X. rasile*).

Collected specimens: 137 in *P*. *isosceles*, 186 in *P*. *patagonicus* and 62 in *X*. *rasile*.

Deposited specimens: CHIOC 39214, 39215, 39216, 39217 (*P*. *isosceles*), CHIOC 39210, 39211a-c (*P*. *patagonicus*), and CHIOC 39212a-c, 39213 (*X*. *rasile*).

Nematoda Pottis, 1932, Chromadorea Inglis, 1893, Chromadoria Pearse, 1942, Rhabditida Chitwood, 1933, Spirurina Railliet & Henry, 1915

Spiruromorpha De Ley & Blaxter, 2002

Camallanoidea Railliet & Henry, 1915

Camallanidae Railliet & Henry, 1915

*Procamallanus* Baylis, 1923

*Procamallanus* (*Spirocamallanus*) Olsen, 1952

*Procamallanus* (*Spirocamallanus*) *halitrophus* (Fusco & Overstreet, 1978) Cárdenas & Lanfredi, 2005

(Figures 5 and 6)

**Figure 5:**
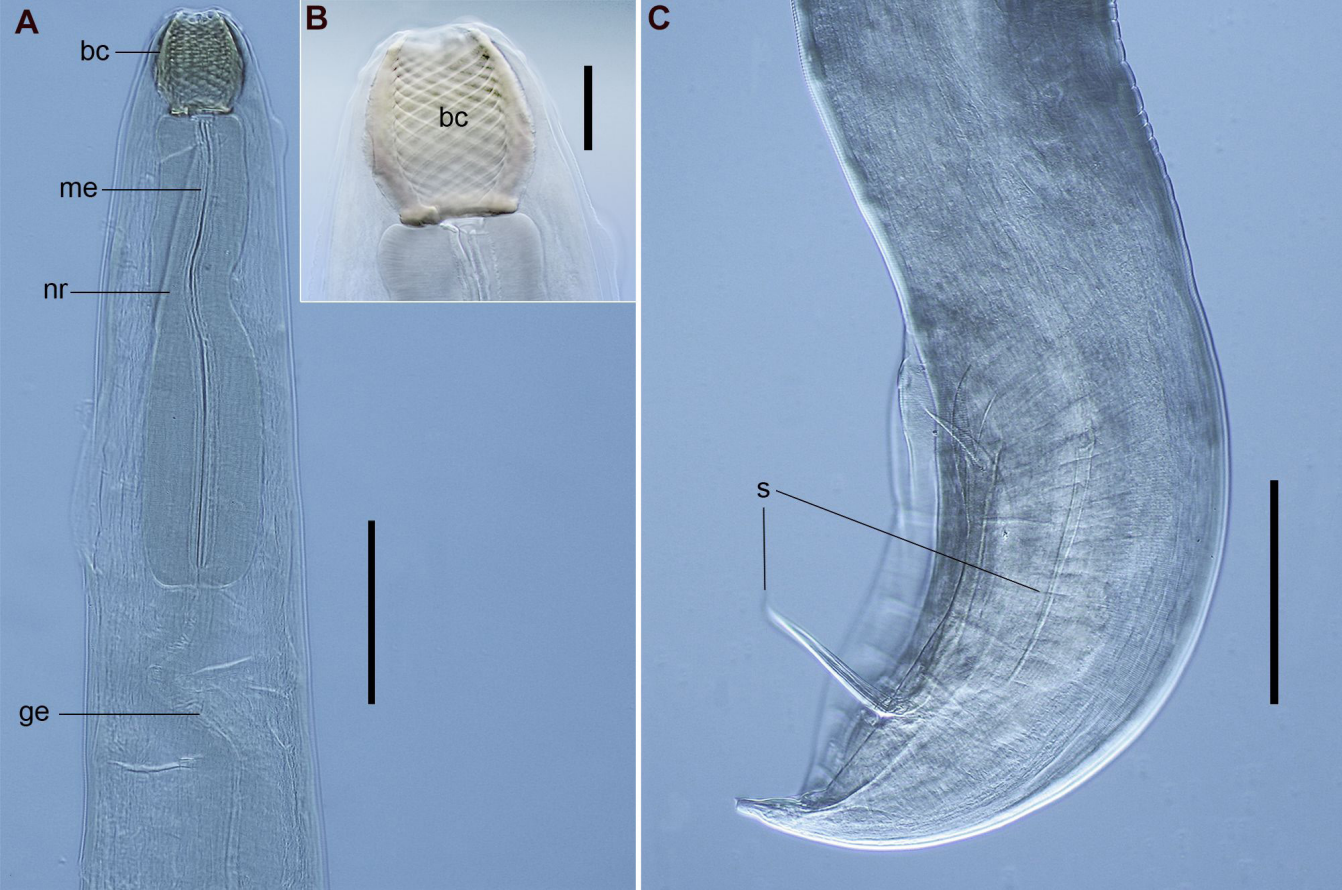
Male of *Procamallanus* (*Spirocamallanus*) *halithrophus* collected from *Paralichthys isosceles*, DIC. (A) Anterior end, lateral view; (B) Detail of buccal capsule; (C) Posterior end, lateral view. Abbreviations: bc = buccal capsule, ge = glandular esophagus, me = muscular esophagus, nr = nerve ring, and s = spicules. Bars A and C = 0.2 mm, B = 0.05 mm.

**Figure 6:**
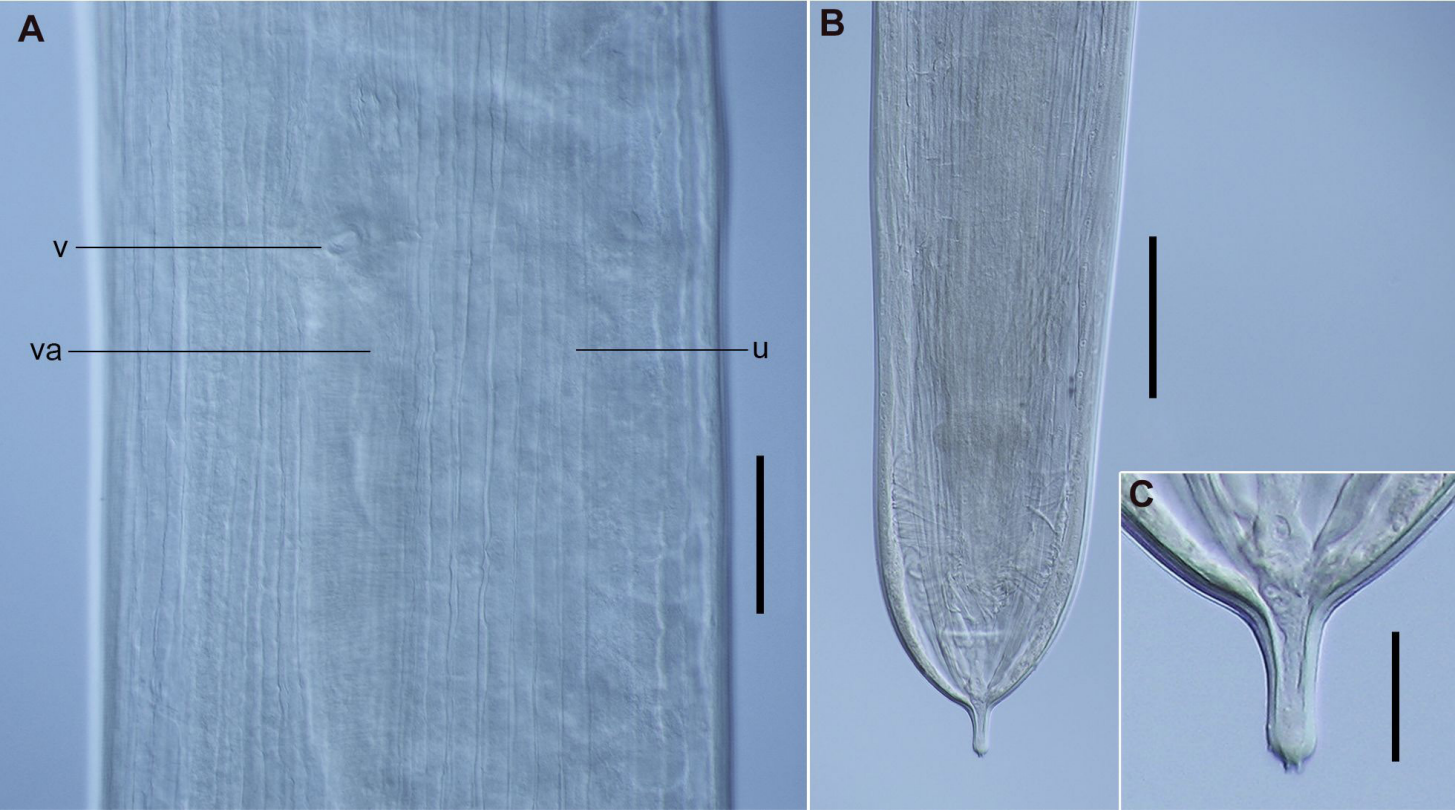
Female of *Procamallanus* (*Spirocamallanus*) *halithrophus* collected from *Paralichthys isosceles*, ventral view, DIC. (A) genital portion of female body; (B) Posterior end; (C) Detail of tail tip showing a terminal digit-like projection with two minute, cuticular spike-like structures. Abreviations: va = vagina, v = vulva, and u = uteri. Bars A = 0.1 mm, B = 0.2 mm, C = 0.05 mm.

Features observed in 20 specimens, 8 from *P*. *isosceles*, and 12 from *X*. *rasile*: Large, reddish and slender nematodes. Both sexes characterized by orange-brown buccal capsule, longer than wide, thick-walled, with basal ring. Inner surface of buccal capsule with cuticular ridges. Muscular region of esophagus claviform, shorter than glandular region. Glandular region ending in paired bilobed valves. Lumen of esophagus triradiate. Oral aperture circular, sometimes rectangular, surrounded by three circlets of four papillae and by two lateral amphids. Outer papillae more prominent than middle papillae. Small deirids situated on lateral line near anterior end, between buccal capsule and nerve ring. Excretory pore ventrally located at anterior portion of body, between muscular and glandular portions of esophagus. Cuticle with deep transversal striations and modified laterally along the body forming the lateral line; striations lacking in cephalic region. Males and females with two terminal cuticular spike-like structures at end of tail, one dorsal and one ventral.

Male: Posterior region with wide caudal alae, ventrally bent and sometimes spiraled. Caudal alae with 11 pairs of transversally elongated papillae, three precloacal, six postcloacal, and two adcloacal, laterally located to cloacal opening. Three pairs of precloacal papillae located ventrally. First and second pairs of postcloacal papillae ventral, third pair lateral, fourth, fifth and sixth pairs dorsal. Cloacal opening longitudinal. Two spicules of unequal length, one covered by a loose sheath, with a pointed distal end.

Female: Body longer than that of males. Muscular vagina opens in vulva, a transversal ventral opening at mid-body. Anus at posterior end with transversal opening. Tail rounded. Pair of phasmids located laterally near tip of tail and anus.

Morphometrics shown in Table 3.

**Table 3:** Morphometric data of *Procamallanus (Spirocamallanus) halitrophus* collected from *Paralichthys isosceles* and *Xystreurys rasile* off the coast of the state of Rio de Janeiro, Brazil.

	Paralichthys isosceles		Xystreurys rasile	
	Males (n = 6)	Females (n = 6)	Males (n = 6)	Females (n = 6)
Body (L)	16.00-20.00 (18.00)	24.6-27.5 (26.17)	16.50-25.90 (21.50)	33.72-39.42 (36.76)
Body (W)	0.16-0.30 (0.25)	0.40-0.50 (0.45)	0.27-0.50 (0.36)	0.60-0.75 (0.66)
Buccal capsule (L)	0.08-0.09 (0.09)	0.10-0.11 (0.10)	0.09-0.11 (0.10)	0.11-0.13 (0.12)
Buccal capsule (W)	0.06-0.09 (0.07)	0.09-0.11 (0.10)	0.08-0.11 (0.09)	0.10-0.11 (0.10)
Esophagus (L)	1.20-1.40 (1.29)	1.34-1.53 (1.42)	1.21-1.40 (1.30)	1.59-2.42 (1.84)
Muscular esophagus (L)	0.40-0.50 (0.45)	0.58-0.63 (0.60)	0.45-0.54 (0.50)	0.64-0.70 (0.67)
Glandular esophagus (L)	0.80-0.90 (0.84)	0.76-0.90 (0.82)	0.71-0.88 (0.80)	0.93-1.73 (1.17)
Nerve ring^a^	0.28-0.33 (0.30)	0.32-0.33 (0.32)	0.26-0.31 (0.29)	0.30-0.40 (0.35)
Vulva^a^	-	12.25-13.80 (13.05)	-	17.30-19.50 (18.42)
Anus^b^	0.16-0.17 (0.16)	0.21-0.25 (0.23)	0.15-0.20 (0.16)	0.22-0.26 (0.24)
Right spicule (L)	0.36-0.45 (0.40)	-	0.31-0.48 (0.40)	-
Left spicule (L)	0.22-0.24 (0.23)	-	0.20-0.29 (0.24)	-

Measurements are in millimeters, means in parentheses. L = length; W = width; n = number of measured specimens;

a distance to the anterior end;

b distance to the posterior end.

Hosts: *P*. *isosceles* and *X*. *rasile.*

Parasitic indices: P = 25.00%, MI = 1.80 (± 1.03), MA = 0.45 (± 1.41), RI = 1-5 (*P*. *isosceles*); P = 30.55%, MI = 1.72 (± 0.90), MA = 0.52 (± 7.07), RI = 1-3 (*X*. *rasile*).

Infection sites: intestine (*P*. *isosceles* and *X*. *rasile*).

Collected specimens: 27 in *P*. *isosceles* and 19 *X*. *rasile*.

Deposited specimens: CHIOC 38732, 38733, 38734, 38735 (*P*. *isosceles*) and CHIOC 38724, 38725, 38726, 38727, 38728, 38729, 38730, 38731 (*X*. *rasile*).

Ascaridomorpha De Ley & Blaxter, 2002, Seuratoidea Hall, 1916

Cucullanidae Cobbold, 1864

Cucullaninae Cobbold, 1864

*Cucullanus* Müller, 1777

**Figure 7:**
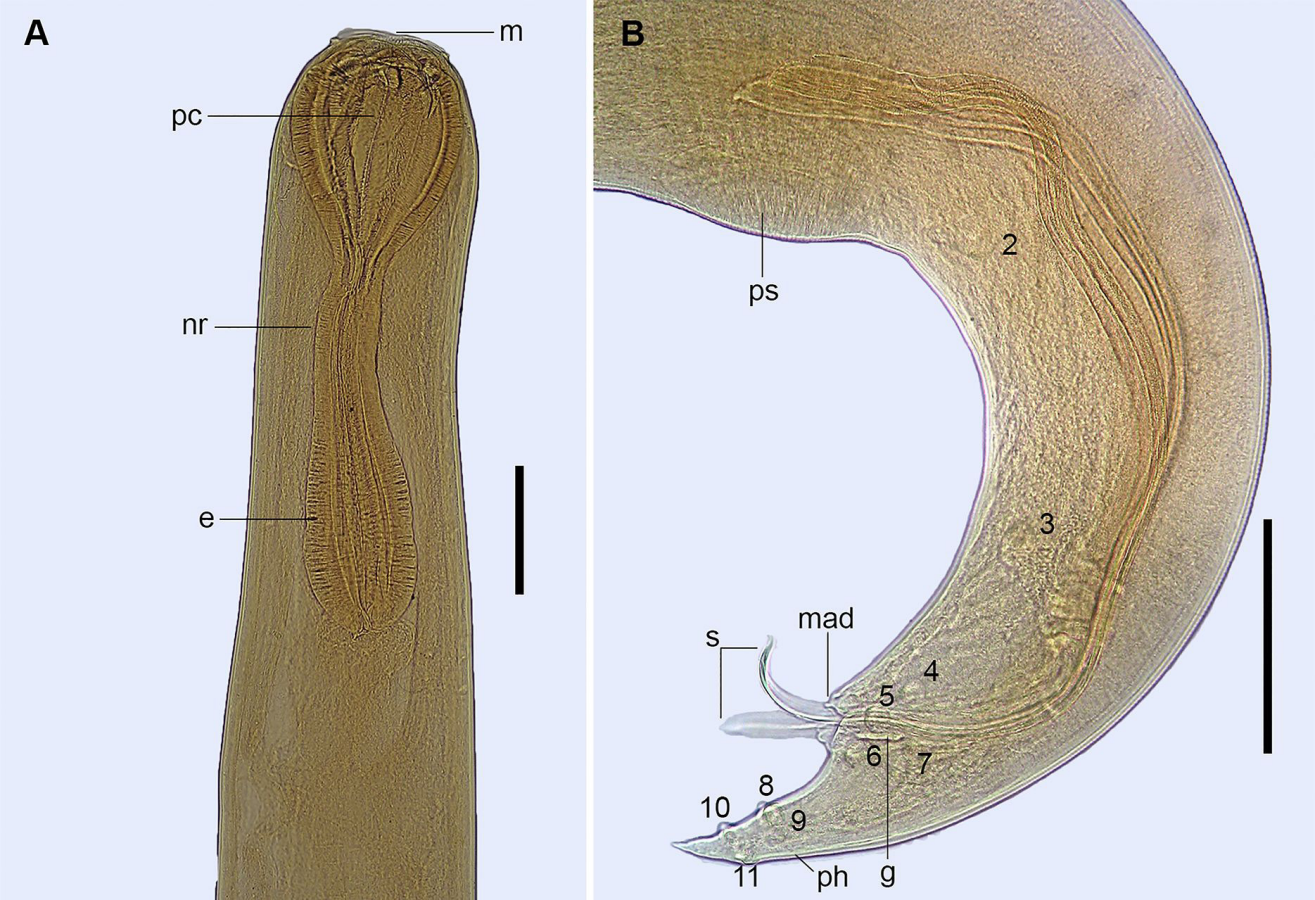
Male of *Cucullanus bonaerensis* collected from *Paralichthys patagonicus*. (A) Anterior end, lateral view; (B) Posterior end, lateral view, visible caudal papillae, precloacal pairs (2, 3), medial adcloacal papilla (mad), adcloacal pairs (4, 5, 6, 7), postcloacal pairs (8, 9, 10, 11) and phasmid pair (ph). Abbreviations: e = esophagus, g = gubernaculum, m = mouth, nr = nerve ring, pc = pseudobuccal capsule, ps = pseudosucker, s = spicules. Bars: A and B = 0.2 mm.

**Figure 8:**
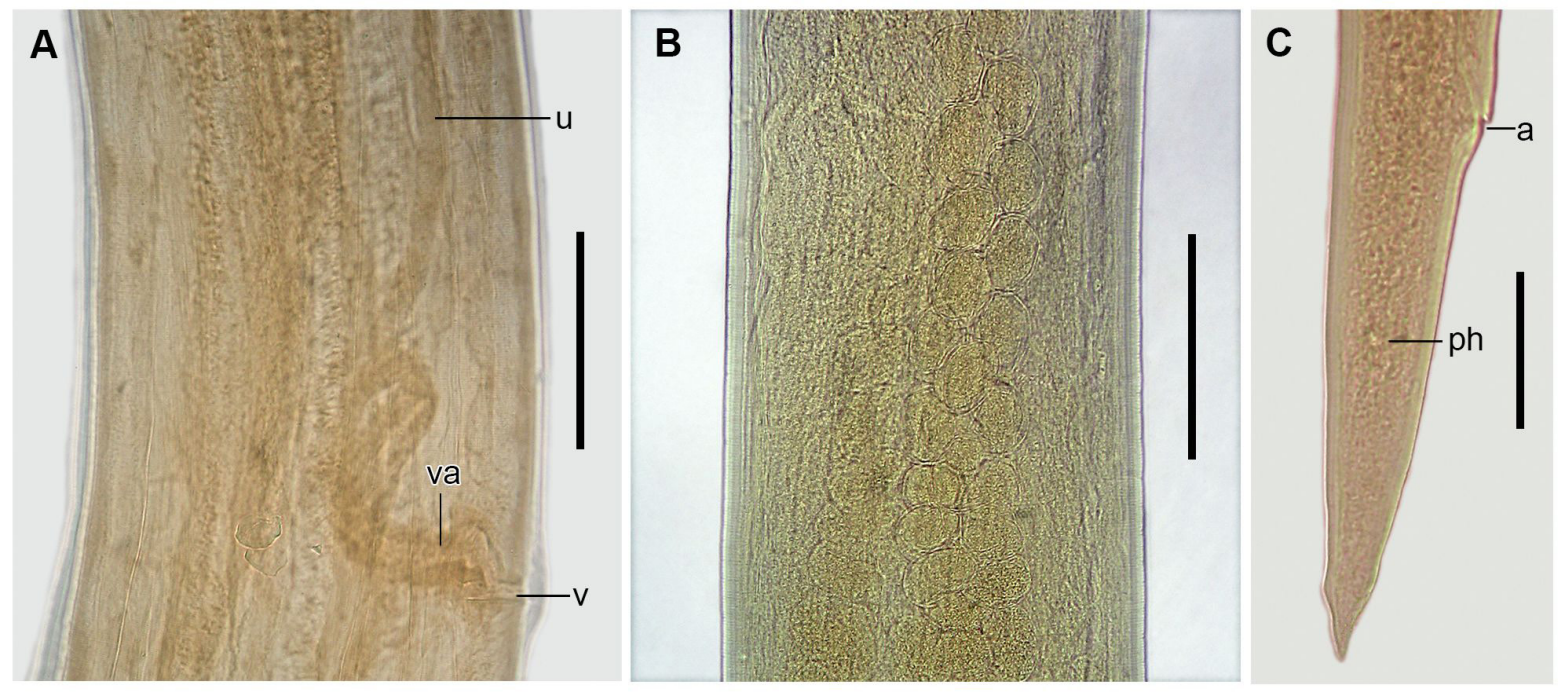
Female of *Cucullanus bonaerensis* collected from *Xystreurys rasile*. (A) Anterior third, lateral view; (B) Eggs inside the uterus, lateral view; (C) Posterior end, lateral view. Abbreviations: a = anus, ph = phasmid, u = uteri, va = vagina, and v = vulva. Bars A and B = 0.2 mm, C = 0.05 mm.

**Figure 9:**
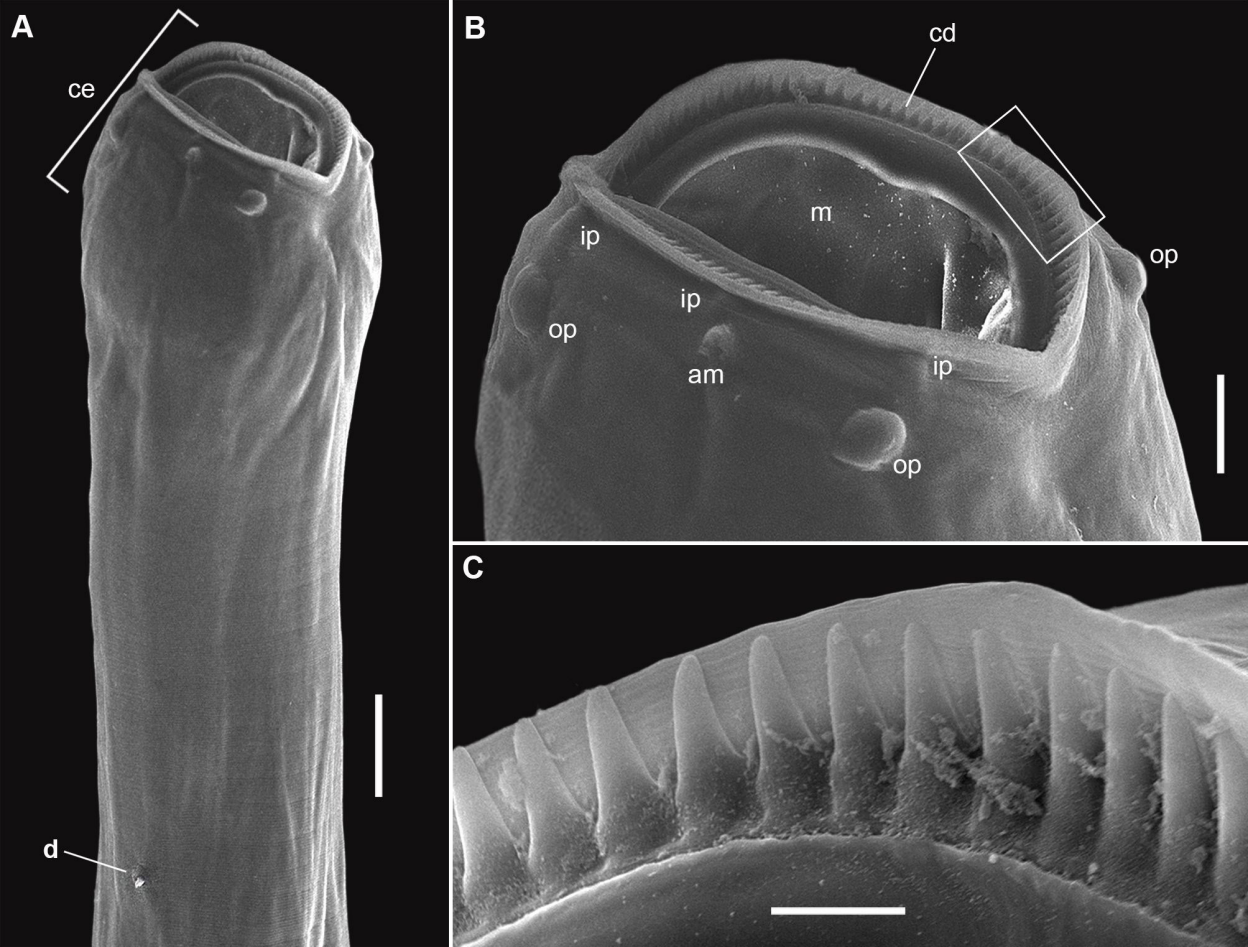
Male of *Cucullanus bonaerensis* collected from *Xystreurys rasile*, latero-ventral view, SEM. (A) Anterior end; (B) Detail of cephalic end; (C) Detail of rectangle of Figure B showing triangular denticles. Abbreviations: am = amphid, ce = cephalic end, cd = collarette of triangular denticles, d = deirid, ip = inner papillae, m = mouth, and op = outer papillae. Bars A = 50 µm; B = 20 µm; C = 5 µm.

**Figure 10:**
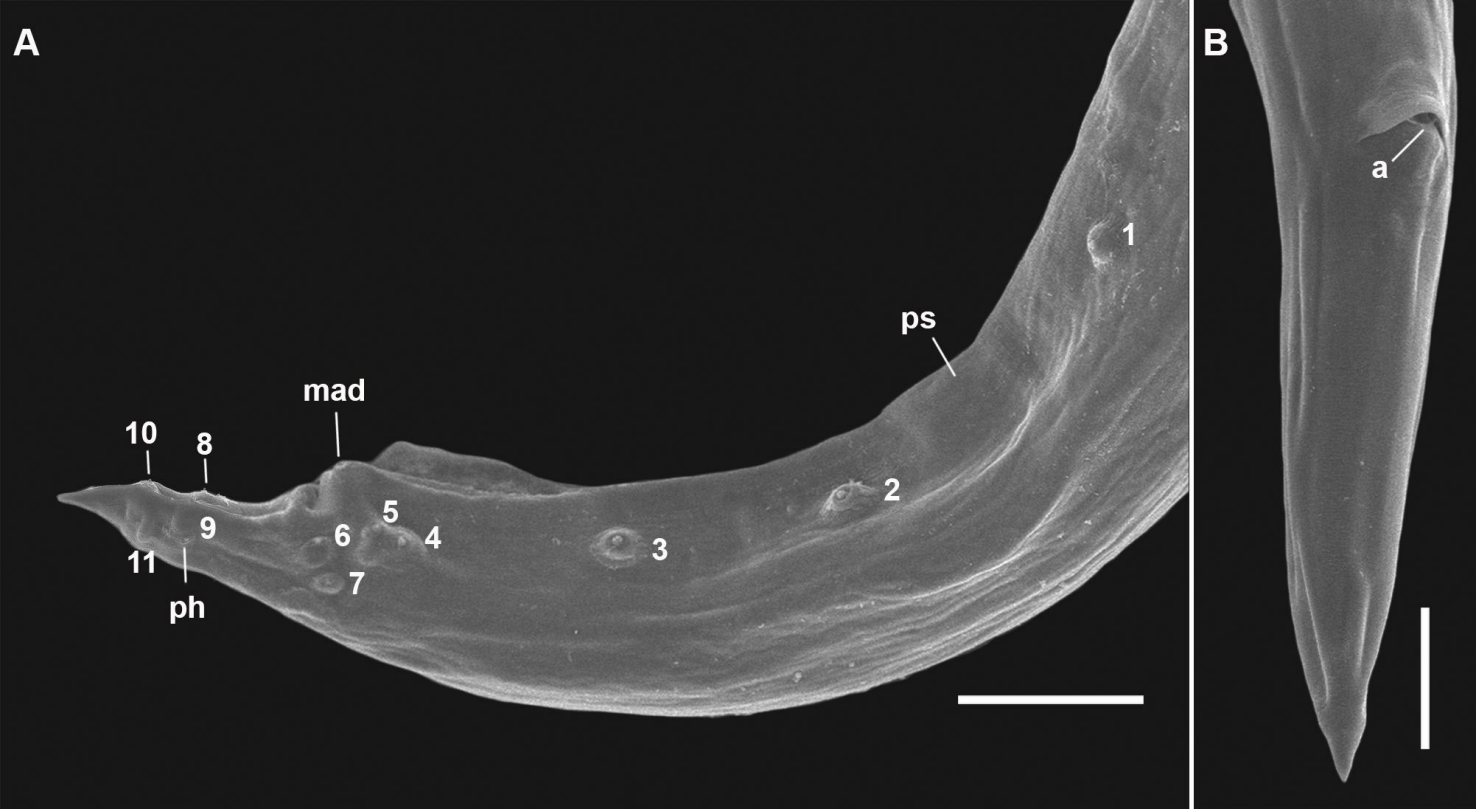
Male and female of *Cucullanus bonaerensis* collected from *Xystreurys rasile*, SEM. (A) Male, posterior end, lateral view showing caudal pseudosucker, caudal papilla pairs 1, 2, 3 (precloacal), 4, 5, 6, 7 (adcloacal), 8, 9, 10, 11 (postcloacal), and medial adcloacal papilla; (B) Female, posterior end, ventrolateral view. Abbreviations: a = anus, mad = medial adcloacal papilla, ph = phasmid and ps = caudal pseudosucker. Bars A = 100 µm, B = 50 µm.

*Cucullanus bonaerensis* Lanfranchi, Timi & Sardella, 2004 (Figures 7-10)

Features observed in 40 specimens, 10 from *P*. *isosceles*, 20 from *P*. *patagonicus* and 10 from *X*. *rasile*: Body slender. Cuticle finely striated throughout. Lateral alae absent. Anterior end rounded, dorsoventrally expanded. Cephalic extremity with usual features of *Cucullanus*, with two pairs of prominent outer papillae, a pair of amphids, and three pairs of small labial inner papillae. Mouth slit-like dorsoventrally, surrounded by collarette armed with numerous triangular denticles. Pseudobuccal cavity well developed with internal cuticular lining, esophagus long and narrow, expanded at both extremities, opening into intestine through small valve; anterior end wider than posterior end. Nerve ring surrounding esophagus at its second third. Deirids and excretory pore situated between the second half and the distal end of esophagus. Right deirid preequatorial, left postequatorial. Tail conical.

Male: Precloacal sucker at posterior body region, between pairs 1 and 2 caudal papillae. Cloaca prominent. Caudal papillae consisting of one medial adcloacal papilla and 11 pairs of papillae, three precloacal pairs (pair 1 anterior and pairs 2 and 3 posterior to ventral sucker; pair 3 closer to cloaca), four adcloacal pairs (pairs 5, 6 and 7 subventral, pair 4 lateral situated slightly anterior to pair 7), and four postcloacal pairs (pairs 9 and 10 subventral, pairs 8 and 11 lateral) and one pair of lateral phasmids, at level of papillae pair 9. Spicules subequal with pointed distal ends. Gubernaculum Y- shaped.

Female: Ovijector directed anteriorly from vulva. Uteri amphidelphic. Eggs fully-developed, oval, thin-walled. Tail with pair of caudal papillae (phasmids) situated at posterior extremity.

Morphometrics shown in Table 4.

**Table 4:** Morphometric data of *Cucullanus bonaerensis* collected from *Paralichthys isosceles*, *P. patagonicus* and *Xystreurys rasile* off the coast of the state of Rio de Janeiro, Brazil.

	Paralichthys isosceles	Paralichthys patagonicus		Xystreurys rasile	
	Females (n = 10)	Males (n = 7)	Females (n = 13)	Male (n =1)	Females (n = 12)
Body (L)	2.59-14.20 (4.99)	2.50-9.37 (6.45)	2.52-9.42 (4.35)	10.20	3.02-12.27 (4.76)
Body (W)	0.17-0.40 (0.18)	0.10-0.45 (0.28)	0.10-0.32 (0.16)	0.30	0.10-0.35 (0.17)
Esophagus (L)	0.36-1.02 (0.55)	0.45-1.18 (0.93)	0.50-1.17 (0.65)	1.14	0.45-1.22 (0.59)
Esophagus (W)	0.09-0.17 (0.10)	0.07-0.09 (0.15)	0.07-0.20 (0.10)	0.16	0.06-0.17 (0.09)
Buccal capsule (L)	0.04-0.23 (0.08)	0.07-0.20 (0.13)	0.05-0.18 (0.08)	1.00	0.03-0.20 (0.08)
Nerve ring^a^	0.19-0.45 (0.20)	0.20-0.50 (0.36)	0.21-0.43 (0.26)	0.40	0.17-0.46 (0.24)
Precloacal sucker^b^	-	0.29-0.86 (0.59)	-	0.89	-
Right spicule (L)	-	0.90-1.04 (0.98)	-	1.40	-
Left spicule (L)	-	0.96-1.11 (1.02)	-	1.10	-
Gubernaculum	-	0.03-0.04 (0.03)	-	0.06	-
Vulva^a^	1.68-7.38 (3.81)	-	2.00-7.12 (3.41)	-	2.10-9.27 (4.44)
Tail^b^	0.12-0.40 (0.19)	0.13-0.21 (0.16)	0.12-0.31 (0.18)	0.16	0.30-0.45 (0.21)
Eggs (L)*	-	-	50.00-90.00 (60.00)	-	-
Eggs (L)*	-	-	30.00-50.00 (40.00)		-

Measurements are in millimeters, means in parentheses. L = length; W = width; n = number of measured specimens;

a distance to the anterior end;

b Distance to the posterior end.

* Measurements in micrometers and number of measured eggs = 50.

Hosts: *P*. *isosceles*, *P*. *patagonicus* and *X*. *rasile.*

Parasitic indices: P = 31.66%, MI = 8.05 (± 8.13), MA = 2.55 (± 12.72), RI = 1-58 (*P*. *isosceles*); P = 41.66%, MI = 7.00 (± 7.69), MA = 2.91 (± 9.89), RI = 1-42 (*P*. *patagonicus*); P = 27.77%, MI = 4.00 (± 3.39), MA = 1.11 (± 6.36), RI = 1-20 (*X*. *rasile*).

Infection site: intestine (*P*. *isosceles*, *P*. *patagonicus* and *X*. *rasile*).

Collected specimens: 153 in *P*. *isosceles*, 105 in *P*. *patagonicus* and 40 in *X*. *rasile*.

Deposited specimens: CHIOC 38745, 38746, 38747, 38748 (*P*. *isosceles*); CHIOC 38736, 38737, 38738, 38739, 38740 (*P*. *patagonicus*) and CHIOC 38741, 38742, 38743, 38744 (*X*. *rasile*).

This study allowed to evidence the first occurrence of parasitism of *P. patagonicus* by *L. monticellii*; *X. rasile* by *A. laguncula* and *P*. (*S*.) *halitrophus*; and *P. isosceles* and *P. patagonicus* by *C. bonaerensis* in the Western South Atlantic Ocean.

## Discussion

The morphology and morphometry of the specimens of *L*. *monticellii* collected in the present study were in accordance with the original description (Linton, 1940) and redescriptions (Skrjabin & Guschanskaja, 1955; Nasir & Díaz, 1971; Fernandes et al., 1985; França et al., 2020) of the species. Morphometrically, the present specimens were smaller than those collected from *Trichiurus lepturus* L. from off the coast of Cumaná, State of Sucre, Venezuela (Nasir & Díaz 1971), and from *Trachurus lathami* Nichols, 1920 and *T. lepturus* from off the coast of the state of Rio de Janeiro, Brazil (Fernandes et al., 1985; França et al., 2020), which can be considered intraspecific variation.

Alarcos & Timi (2012) studied the same three species of flounders as the present study in Argentina and found *L. microstomum* [=*L*. *monticellii*, as suggested by França et al. (2020)] only parasitizing *X*. *rasile*. Alarcos et al. (2016) reported this digenean species parasitizing *P*. *isosceles* from off the state of Rio de Janeiro. The finding of *L*. *monticellii* parasitizing the flounders of the present study expands the knowledge of parasitism of marine teleostean hosts. This digenean has been previously reported occurring off the coast of the Western Atlantic Ocean from northern USA to Nuevo Gulf, Argentine Patagonia (Szidat & Nani, 1951; Nasir & Díaz, 1971; Yamaguti, 1971; Fernandes et al., 1985, 2009; Pereira et al., 2000; Luque et al., 2003, 2008; Cordeiro & Luque, 2005; Kohn et al., 2007; Timi et al., 2010; Carvalho & Luque, 2011; Bueno et al., 2014; Justo & Kohn, 2014, 2015; Pereira et al., 2014; Eiras et al., 2016; França et al., 2020). This is the first report of *L. monticellii* parasitizing *P. isosceles* and *P. patagonicus*.

The morphology and morphometry of the specimens of *A. laguncula* collected in the present study were in accordance with the redescriptions of Manter (1947), Szidat (1961), Nahhas & Short (1965) and Fernandes et al. (1985). Although Bray & MacKenzie (1990) demonstrated the entrance of the metraterm at the base of the sinus sac in an illustration in their redescription of this species, they did not describe this in the text, which is provided by the present study as “metraterm entering sinus sac ventrally joining male duct immediately to the beginning of sinus sac to the internal ejaculatory duct”.

The species *A. laguncula* was found parasitizing *P. patagonicus* in Argentinean waters by Szidat (1961) and in waters off Necochea, Argentina, by Alarcos & Timi (2012), which differs from the present study, which also found it parasitizing *P. isosceles* and *X. rasile*. The species *A*. *laguncula* was found in all three of the flounder species studied, which differs from Alarcos et al. (2016), who reported it only in *P. isosceles* from off the state of Rio de Janeiro, Brazil. Furthermore, the species has been previously reported parasitizing a variety of marine teleostean fish species off Brazil, namely *Balistes capriscus* Gmelin, 1789, *B. vetula* L., *Chaetodipterus faber* (Broussonet, 1782), *Ctenosciaena gracilicirrhus* (Metzelaar, 1919), *Dactylopterus volitans* L., *Micropogonias furnieri* (Desmarest, 1823), *Mullus argentinae* Hubbs & Marini, 1933, *Paralonchurus brasiliensis* (Steindachner, 1875), *Peprilus paru* (L.), *Pseudopercis numida* Miranda Ribeiro, 1903, *Rhomboplites aurorubens* (Cuvier, 1829), *Scomber japonicas* Houttuyn, 1782, *Trachurus lathami* Nichols, 1920, *Umbrina coroides* Cuvier, 1830, and *Urophycis brasiliensis* (Kaup, 1858) (Kohn et al., 2007; Fernandes et al., 2009; Cárdenas et al., 2012a, b; Eiras et al., 2016). Therefore, the list of hosts for *A. laguncula* is expanded to include the flounders studied in the present study. This is the first report of *A. laguncula* parasitizing *P. isosceles* and *X. rasile*.

The morphology and morphometry of the specimens of *P*. (*S*.) *halitrophus* collected in the present study were in accordance with redescription of the species using specimens collected from *Syacium papillosum* L. and *Citharichthys macrops* Dresel, 1889, from off the coast of the state of Rio de Janeiro, Brazil (Cárdenas & Lanfredi, 2005).

In South America, *P*. (*S*.) *halitrophus* has been registered parasitizing some marine teleostean fish off the coast of Brazil, namely *P. isosceles, C. macrops*, *M. argentinae*, *S. papillosum* and *U. brasiliensis* off the states of Rio de Janeiro, Rio Grande do Sul and Santa Catarina (Alarcos et al., 2016; Eiras et al., 2016; Di Azevedo & Iñiguez, 2018). Therefore, this is the first report of *P*. (*S*.) *halitrophus* parasitizing *P. patagonicus* and *X. rasile*.

The morphology and morphometry of the specimens of *C*. *bonaerensis* collected in the present study were in accordance with the description of the species using specimens collected from *Urophycis brasiliensis* (Kaup, 1858) from off the coast of Mar del Plata, Argentina by Lanfranchi et al. (2004).

Alarcos & Timi (2012) found *Cucullanus* sp. and *C*. *bonaerensis* parasitizing *X. rasile* off the Argentine Coast, while the present study found only *C*. *bonaerensis* in the three paralichthyid hosts studied. Alarcos et al. (2016) reported *Cucullanus* sp. in *P. isosceles* from off the coast of the state of Rio de Janeiro, Brazil, which differs from the present study, which found only *C*. *bonaerensis*. Therefore, the present study expands the occurrence of this species in South America. This is the first report of *C. bonaerensis* parasitizing *P. isosceles* and *P. patagonicus*.

Alarcos & Timi (2012) found gravid females of *C*. *bonaerensis* in *X. rasile* from off the Argentine Coast and suggested this species as their definitive host. In the present study, gravid females of this nematode were only found in *P*. *patagonicus*. Lanfranchi et al. (2004) did not find any gravid females of *C*. *bonaerensis*, which they attributed to seasonality in the life cycle of the parasites and/or host suitability.

The sites of infection found here were the same as reported by Alarcos & Timi (2012), stomach for hemiurid and lecithasterid digenean trematodes and intestine for camallanid and cucullanid nematodes.

Comparisons of the parasitic indices of the present study with those for the hemiurid and lecithasterid digeneans and camalanid and cucullanid nematodes collected from flounders in Argentine waters by Alarcos & Timi (2012) found that the latter had a lower prevalence (10.42%) and mean abundance (0.17) for *L*. *microstomum* in *X. rasile*; a lower prevalence (23.53%) and mean abundance (0.94) for *A. laguncula* in *P*. *patagonicus*; and a higher prevalence (39.58%) and lower mean abundance (0.73) for *C*. *bonaerensis* in *X*. *rasile*.

Comparisons with the parasitic indices of helminths parasitizing *P*. *isosceles* off of the state of Rio de Janeiro, Brazil, reported by Alarcos et al. (2016), revealed that these authors found lower prevalences (32.80%, 5.30%,11.10%) and mean abundances (0.95, 0.10, 0.17) for *L*. *microstomum, A. laguncula* and *P*. *halitrophus* (respectively). These differences in parasitic indices could be related to the greater number of collection locations of the present study since, beyond the municipalities of Cabo Frio and Niterói, the present study also collected in the municipalities of Rio de Janeiro and Angra dos Reis. The differences could also be due to various environmental and seasonal factors intrinsic to the collections themselves.

Differences observed in helminthofauna composition and parasitic indices for the same hosts collected in Necochea, Argentina, and Rio de Janeiro, Brazil, could be correlated with the different ecoregions of these areas. According to Spalding et al. (2007), marine ecoregions comprise relatively homogeneous sets of species that are clearly distinct from adjacent systems. The dominant biogeographic agents that define ecoregions vary from location to location but may include physico-chemical and biological factors. Thereby, features observed within ecoregions can influence their fish parasite communities and, thus, explain the parasite compositions found for flounder species from waters off Argentina by Alarcos & Timi (2012) and those found for the same flounder species from waters off of the state of Rio de Janeiro, Brazil, by the present study and for that found for *P. isosceles* by Alarcos et al. (2016).
